# HIV-1 Vpr induces an NFAT-controlled transcriptional program in primary CD4+ T cells

**DOI:** 10.1128/mbio.03605-25

**Published:** 2026-02-04

**Authors:** Johanna Leyens, Carlos Alberto Vanegas-Torres, Anthea Darius, Rabea Seizer, Brigitta Maurer, Daniel Sauter, Rishikesh Lotke, Michael Schindler

**Affiliations:** 1 Institute for Medical Virology and Epidemiology of Viral Diseases, University Hospital Tübingen 27203 , Tübingen, Germany; The University of North Carolina at Chapel Hill, Chapel Hill, North Carolina, USA

**Keywords:** HIV-1, Vpr, NFAT, transcriptional network, G2 arrest, cell cycle, CD4+ T cells

## Abstract

HIV-1 Vpr has a major impact on the cellular transcriptome and proteome. Although Vpr is known to stimulate the nuclear factor of activated T cells (NFAT), it remained unclear to what extent transcriptional changes induced by Vpr are governed by NFAT. We, therefore, performed RNA sequencing and transcription factor network analyses of primary CD4+ T cells infected with HIV-1 harboring an intact or defective *vpr* open reading frame. Furthermore, we analyzed Vprs from HIV-1 groups M, N, O, and P to investigate whether Vpr-mediated NFAT activation is conserved among different clades of HIV-1. All Vprs stimulated NFAT and induced the expression of the T cell activation marker CD69, which is a bona fide target of NFAT in T cells. Our transcriptome analysis showed that NFAT controls 46.5% of 1,083 Vpr-deregulated genes in primary CD4+ T cells. Gene set enrichment analyses revealed that Vpr upregulates processes related to signaling, proliferation, and immunity, while downregulating cell cycle progression, ribosome activity, and cytoskeleton organization. Quantitative real-time PCR confirmed Vpr-mediated modulation of specific genes, i.e., upregulation of *NEIL1, TNFS4,* and *CXCL10*, as well as downregulation of *CCNB1, CDC20, CENPA,* and *PLK1*. Notably, NFAT inhibition abrogated Vpr-mediated enhancement of HIV-1 replication in primary CD4+ T cells and alleviated G2 arrest in Jurkat T cells. In conclusion, a significant proportion of Vpr-deregulated genes in CD4+ T cells are controlled by NFAT. Affected pathways are related to T cell activation, cell cycle progression, cytoskeleton, and chromosome organization. Thus, Vpr-mediated NFAT activation is a key regulatory event that reprograms the host cell transcriptome into an environment supportive of HIV-1 replication.

**IMPORTANCE:**

The HIV-1 accessory protein Vpr is known for its profound effect on the host proteome. It degrades many cellular proteins, including transcription factors and DNA-associated proteins. In addition, Vpr activates the nuclear factor of activated T cells (NFAT), a key transcription factor in T cells. However, it has remained unclear to what extent Vpr and consequently NFAT control changes in the transcriptome of HIV-1-infected primary CD4+ T cells. In this study, we show that Vpr significantly alters the transcriptome of CD4+ T cells, with almost half of the deregulated genes being under NFAT control. These changes involve pathways associated with increased immune activation and cell cycle regulation, shedding light on how Vpr contributes to CD4+ T cell depletion and HIV-1 pathogenesis.

## INTRODUCTION

Vpr is a small lentiviral ~96 aa accessory protein with a size of 12–14 kDa that is incorporated into HIV-1 particles. Given its conserved appearance within the HIV-1 genome and its importance for the pathogenicity of HIV-1 and SIV *in vivo*, it is clear that Vpr fulfills essential functions within the host cell, likely related to antiviral immune evasion and viral replicative fitness (1–3). Nevertheless, the mechanisms and exact functionalities explaining why Vpr is important for pathogenicity and high viral loads *in vivo* have remained unclear.

Vpr acts as an adaptor protein that recruits individual target proteins to DCAF1 (DDB1- and CUL4-associated factor 1), which is also known as Vpr-binding protein (4, 5). DCAF1 acts as a substrate recognition receptor, linking target proteins of Vpr to the E3-ubiquitin ligase complex CUL4A and ultimately mediating their proteasomal degradation. Examples include EXO1 (6) and HLTF helicases (7), as well as the DNA damage response-associated proteins UNG2 (8, 9), MUS81, and EME1 (10), highlighting Vpr’s role as a deregulator of the host cell proteome (11–13). Another remarkable feature of Vpr is that its expression—either alone or in the context of the viral genome—induces a G2 cell cycle arrest in several cell lines, including HEK293 derivatives, HeLa cells, and some immortalized CD4+ T cell lines such as Jurkat or SupT1 cells (5, 14–25). Of note, even though this G2 arrest is widely accepted as a hallmark function of Vpr, it remains an enigma why a virion-incorporated small accessory protein should arrest cells in G2, likely early after infection, before the onset of HIV-1 gene expression. One hypothesis is that the HIV-1 LTR has a higher transcriptional activity in G2-arrested cells (26). Notably, mutation of position Q65 not only abrogates the ability of Vpr to induce a G2 arrest but also disrupts its interaction with DCAF1, suggesting that both functions are genetically linked (11, 27). In line with this, depletion of factors that are degraded by Vpr via DCAF1 can result in a G2 arrest. Examples include MCM2 (13), the exonuclease EXO1 (exonuclease 1) (6), and CCDC137 (24). These findings resulted in the dogma that Vpr recruits and degrades a specific set of host cell factors in a DCAF1-E3-CUL4A-dependent manner in order to arrest infected cells in the G2 phase of the cell cycle (24, 25, 28). However, to the best of our knowledge, compelling evidence for Vpr-induced G2 arrest in primary CD4+ T cells is sparse and limited to highly and unphysiologically stimulated CD4+ T cells (29, 30). Of note, in recent years, other viral accessory proteins have also been associated with altered cellular proliferation and cell cycle. HIV-1 Vpu degrades the amino acid transporter SNAT1 to manipulate host cell metabolism (31), while Vif has been firmly linked to degrade PP2A, causing cell cycle arrest in several cell lines (32–34).

HIV-1 Vpr is also known to stimulate nuclear factor of activated T cells (NFAT) activation (21, 35, 36). To date, five NFAT gene family members have been identified. These are differentially expressed in various cell types and tissues (37). Four of them, termed NFATc1–NFATc4, are canonically activated via Ca^2+^ that triggers the phosphatase calcineurin to dephosphorylate cytoplasmic pNFATc1-c4. Upon dephosphorylation, these NFATs shuttle into the nucleus to either enhance or suppress the expression of their target genes. In contrast, NFAT5 is non-canonically activated without the involvement of Ca^2+^ and calcineurin. NFATc1 is considered a key transcription factor, playing a crucial role during T activation, differentiation, and function (38, 39). Besides their key role in lymphocyte physiology, members of the NFAT family exert multiple additional regulatory functions and are even expressed in a variety of non-hematopoietic cells, including neurons, muscle cells, and kidney epithelial cells (37, 40).

Given the role of NFAT family members in regulating gene expression networks in various lymphocyte signaling pathways (41), NFAT activity is believed to be an important determinant of the permissiveness of the host cell to HIV-1. In line with this, previous work of our group demonstrated that virion-delivered Vpr induces the activation and nuclear translocation of NFAT, thereby supporting productive HIV-1 infection. Furthermore, we found that NFAT activation correlates with Vpr-mediated LTR transactivation and G2 arrest in T cell lines (21).

It is conceivable that NFAT activation and DCAF-mediated degradation of host cell factors both have a major impact on the cellular transcriptome. Here, we therefore performed RNA sequencing (RNA-seq) analyses of HIV-1-infected primary CD4+ T cells to decipher the relative contribution of altered NFAT activity to Vpr-mediated modulation of host cell transcription. Furthermore, we investigated a potential role of NFAT activation in G2 arrest and analyzed the evolutionary conservation of NFAT activation among diverse HIV-1 Vprs.

## MATERIALS AND METHODS

### Plasmids and proviral constructs

HIV-1 constructs based on the pBR NL4-3 backbone, including those with intact or deleted *vpr* gene, were used as described previously (21, 42, 43). GFP-expressing pWPI lentiviral vectors encoding various primary HIV-1 Vprs, along with their respective controls, were kindly contributed by Éric A. Cohen (Montreal, Canada) (44). Packaging plasmid psPAX2 and envelope plasmids pVSV-G (pMD2G) were obtained from Addgene.

### Cell lines and primary CD4+ T cells

HEK293T cells were cultured in Dulbecco’s Modified Eagle Medium (Gibco) containing 10% heat-inactivated fetal calf serum (FCS; Gibco), 1% penicillin/streptomycin (P/S; Life Technologies), and maintained at 37°C in a 5% CO_2_ atmosphere. Jurkat E6.1, Jurkat-NFAT-Luc, and Jurkat-Dual cells were cultured in RPMI-1640 (Life Technologies), supplemented with 10% heat-inactivated FCS (Gibco) and 1% P/S (Life Technologies); Jurkat-Dual additionally received 2 mM L-glutamine (Merck Millipore), 10 μg/mL Blasticidin, and 100 μg/mL Zeocin (InvivoGen). Primary CD4+ T cells were isolated from buffy coats (ZKT Tübingen gGmbH, Tübingen, Germany). Donors provided written informed consent. CD4+ T cells were isolated by negative selection using the RosetteSep CD4+ T cell isolation kit (StemCell Technologies). Isolated CD4+ T cells were cultured in RPMI-1640 medium supplemented with 10% FCS, 2 mM L-glutamine, and 10 ng/mL IL-2 (StemCell Technologies). Depending on the experimental setup, cells were either pre-stimulated with 1 µg/mL phytohemagglutinin (PHA; Thermo Fisher Scientific) for 3 days before infection, stimulated over the TCR with 2 µg/mL plate-bound anti-CD3 (clone HIT3a, Biolegend) plus 1 µg/mL soluble anti-CD28 (clone CD28.2, Biolegend) for 24 h before infection, or directly infected with HIV-1.

### Generation of HIV-1 virions and lentiviral particles

HEK293T cells were seeded in 6-well plates (4.5 × 10^5^ cells/well) 24 h before transfection. HIV-1 viral stocks were produced by transfecting HEK293T cells with the proviral vectors, employing the JetPRIME transfection kit in accordance with the manufacturer’s instructions (Polyplus). These cells received a medium change 4 h post-transfection, and virus-containing supernatants were harvested 28 h thereafter. VSV-G-pseudotyped HIV-1 viral stocks were generated in a similar manner, with cells being co-transfected with proviral vector and pVSV-G expression plasmid (pHIT_VSV-G) (45) at a ratio of 10:1 using the Ca_3_(PO_4_)_2_ method. These cells received a medium change at 16 h post-transfection, and viral stocks were harvested 32 h thereafter. Vpr-expressing VLPs were generated as described beforehand (23, 43, 44). Viral stocks were centrifuged at 3,200 × *g* for 10 min in order to clarify them from remaining cells and debris, and depending on the experiment, VSV-G-pseudotyped HIV-1 NL-4-3 viral stocks underwent an additional concentration step, where the corresponding supernatants were overlaid on 20% sucrose and centrifuged between 90 min and 4 h at 20,000 × *g* and 4 °C. Viral pellets were resuspended in FCS-free RPMI-1640 medium.

### p24 antigen quantification

To quantify the p24 antigen, virus-containing samples were inactivated by incubation with PBS containing 10% Triton X-100 (Merck Millipore) for 30 min at 37°C. p24 concentration was analyzed with an in-house developed p24-ELISA (46). The absorbance was read using a Berthold TriStar2 S LB 942 multimode reader (Berthold Technologies GmbH & Co. KG).

### Lentiviral transduction and infection assays

Jurkat E6.1, Jurkat-NFAT-Luc, and Jurkat-Dual cells were seeded at 7.5 × 10^5^ cells/mL and transduced with VLPs expressing Vpr. Jurkat E6.1 cells were harvested 48 h post-transduction for flow cytometry (CD69 and cell cycle/PI) and western blot (Vpr detection). Jurkat-NFAT-Luc cells were additionally stimulated with 1 µg/mL PHA 8 h before harvest, and at 32 h post-transduction, cells were harvested for intracellular luciferase and stained for CD69 expression and analyzed by flow cytometry. Jurkat-Dual supernatants were collected at 32 h post-transduction and stored at −20°C for secreted luciferase assays. Jurkat E6.1 cells were infected at 1 × 10^6^ cells/mL with 50 ng p24/mL, using equal p24 inputs for HIV-1 and Δ*vpr* stocks. Where indicated, cultures received 10 µM nevirapine (Merck) at the time of infection with DMSO as a vehicle control. Cultures were washed 6 h post-infection, replenished with fresh medium (containing nevirapine where indicated), and maintained for up to 48 h. Cells were then harvested for flow cytometry (infection rate, CD69 expression, and cell cycle/PI). Primary CD4^+^ T cells were infected at 2–3 × 10^6^ cells/mL with 200–300 ng p24/mL, using equal p24 inputs for HIV-1 and HIV-1 Δ*vpr*. Infection was enhanced by spinoculation (800 × *g* for 45 min at 37°C), cultures were washed 6 h post-infection, and cells were resuspended in RPMI-1640 containing IL-2. Infections were followed for up to 96 h, with supernatants and cells collected every 24 h for p24 ELISA and flow cytometry (infection rate and cell cycle/PI), respectively.

### NFAT inhibition

To inhibit NFAT signaling, Jurkat E6.1 cells and primary CD4^+^ T cells were pre-treated with 10–100 ng/mL FK-506 (Invitrogen) or 2.5 µM INCA-6 (MedChemExpress) 2 h before lentiviral transduction or HIV-1 infection. Inhibitors were maintained in culture until harvest. DMSO served as the vehicle control (final ≤0.1% [vol/vol]).

### NFAT knockdown

NFAT1–NFAT4 small interfering RNA targets were described previously (47) and synthesized as custom ON-TARGETplus siRNAs (Horizon/Dharmacon). Target sequences were NFAT1 5′-AUGGAUUCUGGAGCCGAGUUUCUCC-3′, NFAT2 5′-AAACUGGUUAUUGUUGUGGUACAGG-3′, NFAT3 5′-UCAGUGGCACCAAGGUGUUGGAGAU-3′, and NFAT4 5′-GCACAUGAAGAUGACCUACAGAUAA-3′. A non-targeting ON-TARGETplus siRNA (Horizon/Dharmacon) served as a negative control. For siRNA-mediated NFAT knockdown via transient transfection by electroporation, Jurkat E6.1 cells were washed once in PBS and resuspended in resuspension buffer R (Neon, Thermo Fisher). Per reaction, 2 × 10^6^ cells were mixed in a 100 µL Neon tip with 10 µL of 20 µM siRNA (final 2 µM). Electroporation was performed on a Neon Transfection System (Thermo Fisher Scientific) with 3 pulses, 10 ms, and 1,600 V. Cells were transferred immediately into pre-warmed RPMI-1640 without antibiotics. Six hours post-electroporation, the medium was replaced with RPMI-1640 + 10% FCS + 1% P/S. At 48 h after electroporation, cells were counted and infected as described above. At 48 h post-infection, cells were harvested for flow cytometry (infection rate, CD69 expression, and cell cycle/PI) and western blot (NFAT protein levels).

### RNA sequencing

Total RNA of primary PHA pre-stimulated and infected CD4+ T cells was extracted at 48 hpi using the RNeasy Mini Kit (Qiagen) according to the manufacturer’s instructions. RNA homogenization was enhanced using QIAshredder spin columns (Qiagen), and DNA contamination was minimized by on-column DNase digestion (Qiagen). RNA samples were eluted twice with 35 μL RNase-free water, quantified using a NanoDrop spectrophotometer (Thermo Fisher Scientific), and stored either at −20°C (short term) or −80°C (long term). RNA samples were transferred to the c.ATG core facility (Tübingen, Germany) for sequencing.

### Quantitative real-time PCR

Total RNA from primary pre-stimulated and infected CD4+ T cells was extracted at 48 hpi and reverse transcribed using the QuantiTect Reverse Transcription Kit (Qiagen). RT-qPCR was conducted in triplicate for housekeeping and target genes using the Luna Universal qPCR Master Mix (New England Biolabs). Each reaction included 5 ng of cDNA and 6 pmol of QuantiTect Primers (Qiagen). QuantiTect Primer Assays (Qiagen) used are as follows: NEIL1 (Hs_NEIL1_1_SG; QT00493794), TNFSF4 (Hs_TNFSF4_1_SG; QT00028658), CXCL10 (Hs_CXCL10_1_SG; QT01003065), ZBP1 (Hs_ZBP1_1_SG; QT00029631), CD70 (Hs_CD70_1_SG; QT00998340), PTK2 (Hs_PTK2_1_SG; QT00057687), RCBTB2 (Hs_RCBTB2_1_SG; QT00007021), IL18RAP (Hs_IL18RAP_1_SG; QT00036393), CCNB1 (Hs_CCNB1_1_SG; QT00006615), DUSP4 (Hs_DUSP4_1; QT00067018), CDC20 (Hs_CDC20_1_SG; QT00089558), NEK2 (Hs_NEK2_2_SG; QT01668394), IL13 (Hs_IL13_1_SG; QT00000511), CENPA (Hs_CENPA_1_SG; QT00001057), DUSP2 (Hs_DUSP2_1_SG; QT00199794), CDKN3 (Hs_CDKN3_1_SG; QT00014728), PLK1 (Hs_PLK1_1_SG; QT00049749), and CKS2 (Hs_CKS2_1_SG; QT00014693). Reactions were run on a LightCycler 480 (Roche) with an initial denaturation at 95°C for 10 min, followed by 45 cycles of 95°C for 10 s, 55°C for 15 s, and 72°C for 15 s, with fluorescence detection at 522 nm. Relative gene expression was calculated using the 2-^ΔΔ^Ct method (48).

### Flow cytometry

Paraformaldehyde (PFA)-fixed cells were washed with FACS buffer (PBS with 1% FCS) before staining. For surface staining, cells were incubated with anti-human CD69 (clone FN50; PE or BV421; BioLegend) at 1:50 in FACS buffer for 30 min at 4°C and then washed. For intracellular staining, cells were incubated with anti-HIV-1 core antigen (RD1 or FITC; Beckman Coulter) at 1:100 in 0.1% Triton X-100 in PBS for 30 min at 4°C and then washed with FACS buffer. Alternatively, cells were permeabilized and stained using the eBioscience FoxP3/Transcription Factor Staining Buffer Set (Thermo Fisher Scientific) according to the manufacturer’s instructions using the antibody dilutions described above. Stained cells were resuspended in FACS buffer and immediately analyzed.

### Measurement of G2 arrest

To assess G2/M arrest in infected primary CD4+ T cells and Jurkat E.6 T cells, cells were additionally stained with propidium iodide (PI) solution (50 µg/mL PI, 0.1% sodium citrate, 0.1% Triton X-100, and 0.33 mg/mL RNase A in PBS). After 30 min of incubation at room temperature, cells were immediately analyzed for cell cycle distribution using a MACS Quant Analyzer (Miltenyi Biotec).

### Luciferase assays

Luciferase activity in Jurkat-Dual cell supernatants was measured using QUANTI-Luc 4 Lucia/Gaussia Detection Reagent (InvivoGen) according to the manufacturer’s protocol. For Jurkat-NFAT-Luc cells, cell pellets were lysed using a homemade cell lysis buffer (100 mM KH_2_PO_4_, 1% Triton X-100, and 1 mM DTT, pH 7.8) for 10 min at room temperature. The lysate was transferred to a white 96-well plate and mixed 1:1:1 with luciferase assay buffer (100 mM KH_2_PO_4_, 15 mM MgSO_4_, and 5 mM ATP, pH 7.8) and luciferase substrate buffer (1 mM D-luciferin in luciferase assay buffer). Luminescence was measured using a Cytation3 instrument (BioTek Instruments Inc.).

### Western blotting

To assess NFAT and Vpr expression, total protein from Jurkat E6.1 cells was lysed using RIPA buffer supplemented with protease inhibitor (SIGMAFAST Protease Inhibitor; Sigma-Aldrich) and phosphatase inhibitor (PhosSTOP; Merck). Proteins were resolved on 10% Tris–glycine gels and transferred to nitrocellulose (0.2 µm; VWR) by wet transfer (Tris–glycine, 20% methanol, 4°C). Membranes were blocked in 5% (wt/vol) skim milk in TBS for 1 h at room temperature, incubated overnight at 4°C with primary antibodies in blocking buffer + 0.1% Triton X-100, and then probed with IRDye 680RD/800CW-conjugated secondary antibody in blocking buffer + 0.1% Triton X-100 for 1 h at room temperature. Blots were visualized on the LI-COR Odyssey infrared imaging system, and band pixel intensity was quantified using Odyssey (LI-COR). Membranes were subsequently reprobed with anti-GAPDH as a loading control. The following antibodies were used: rabbit anti-NFAT1 (1:1,000; Cell Signaling Technology), rabbit anti-NFAT2 (D15F1) (1:1,000; Cell Signaling Technology), rabbit anti-NFAT3 (23E6) (1:1,000; Cell Signaling Technology), rabbit anti-NFAT4 (1:1,000; Cell Signaling Technology), HIV-1 Vpr (1–50) antiserum (1:800; NIH HIV Reagent Program, Division of AIDS, NIAID #11836, contributed by Dr. Jeffrey Kopp), rat anti-GAPDH (1:1,000; BioLegend), and IRDye 680RD- or 800CW-conjugated anti-rabbit and anti-rat IgG secondary antibodies (1:15,000; LI-COR).

### Software and analyses

Unprocessed RNA-seq data were uploaded to the Galaxy web platform, and the public server at https://usegalaxy.org/ was used to perform differential expression analyses employing the DESeq2 (version 1.40.2) package therein (49). The resulting data were manually curated as described within this publication and used as input for gene ontology enrichment analysis using enrichGO within clusterProfiler (version 4.10.1). Flow cytometric analyses were performed using FlowLogic (version 8.3). Luminescence data were processed using Gen5 (version 3.12). Absorbance data (for p24 quantification) were processed with ICE (version 1.0.9.8). RT-qPCR data were processed using the LightCycler 480 Software (version 1.5.1.62). Statistical analyses were performed using GraphPad Prism (version 10.1.1) and Microsoft Excel. Figures were generated using GraphPad Prism (version 10.1.1), CorelDRAW 2024 (version 25.0.0.230), ggplot2 (version 3.5.0), EnhancedVolcano (version 1.20.0), enrichplot (version 1.22.0), Image Studio Lite (version 5.2.5), as well as with https://www.biorender.com/, Microsoft PowerPoint, and Microsoft Excel.

## RESULTS

### NFAT activation is a conserved function of Vprs derived from HIV-1 M, N, O, and P

We previously showed that virion-delivered Vpr is sufficient to activate NFAT and induce transcription of an NFAT-dependent reporter in Jurkat T cells (21). However, these experiments were performed with cell culture-adapted HIV-1 NL4-3 (group M, subtype B), and it has remained unclear if Vprs from other HIV-1 groups and subtypes also modulate NFAT. To address this, we took advantage of a collection of primary HIV-1 Vprs that are expressed from a lentiviral vector, co-expressing GFP as an infection marker (43, 44).

**Fig 1:**
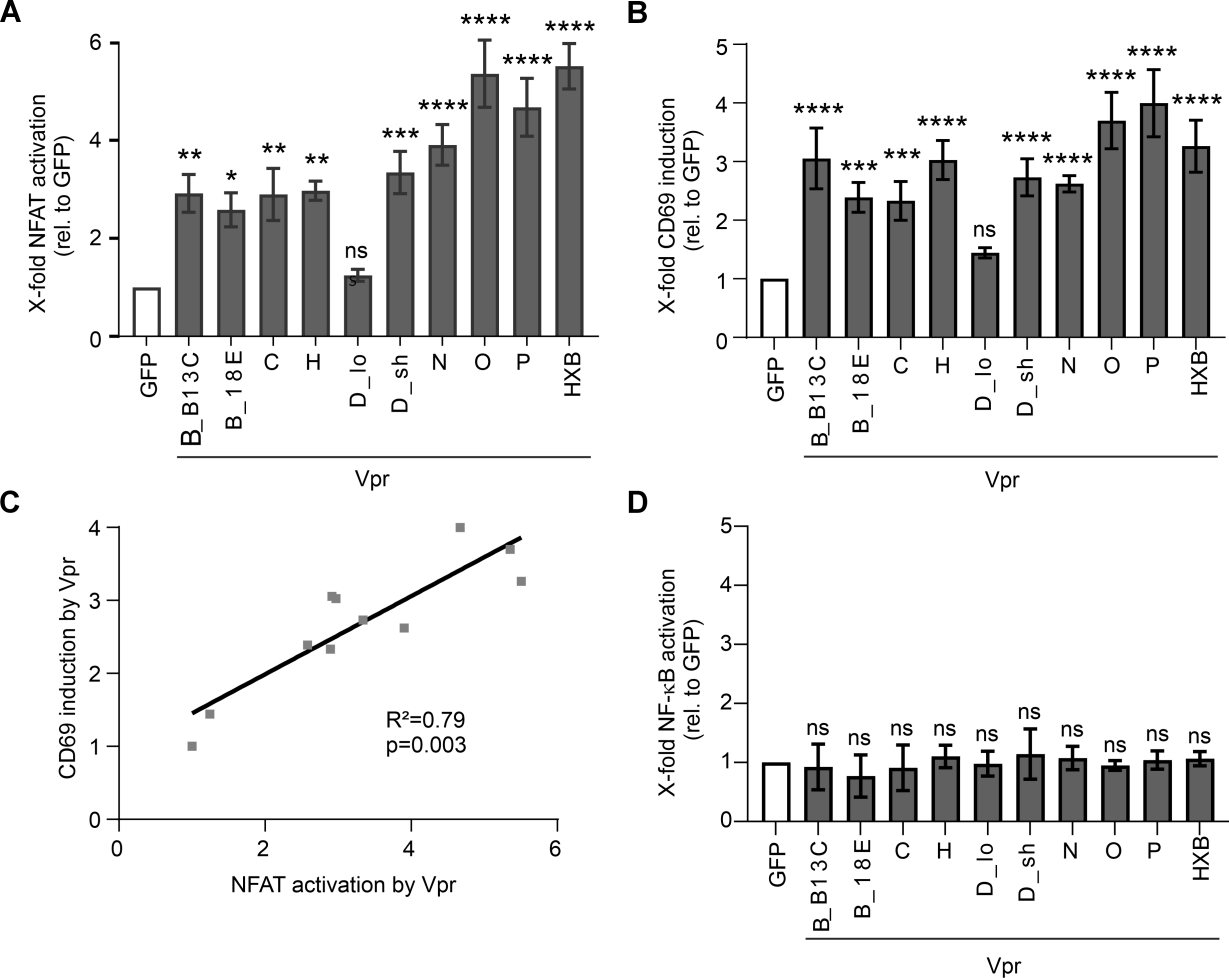
NFAT but not NF-kB activation is a conserved function of Vpr. (**A**) Jurkat-NFAT-luc cells were transduced with VLPs expressing the indicated Vprs. Eight hours before harvest, cells were stimulated with PHA (1 µg/mL), and 32 h post-infection, NFAT-dependent luciferase activity was measured, (**B**) along with CD69 protein levels at the cell surface by flow cytometry; *n* = 4 (mean ± SEM). (**C**) Linear regression indicating a positive correlation between Vpr-mediated NFAT activation and CD69 upregulation. (**D**) Jurkat-dual cells were transduced as described above, and supernatants were collected 32 h post-transfection to analyze NF-κB-dependent luciferase reporter gene expression; *n* = 3 (mean ± SEM). Statistical significance was calculated using one-way ANOVAs coupled to Šidák’s multiple comparisons test. α = 0.05; *P < 0.05; **P < 0.01; ***P < 0.001; ****P < 0.0001; and ns, not significant.

We used Jurkat-NFAT cells that express luciferase under the control of an NFAT-dependent promoter and therefore allow for a rapid and reliable readout of NFAT activity. When Jurkat-NFAT cells were infected with the Vpr-expressing lentiviruses and PHA-stimulated to enhance NFAT activation, we observed three- to sixfold higher activity of NFAT compared to cells transduced with a lentivirus expressing GFP only (Fig. 1A). Vprs from HIV-1 M subtypes B, C, D, and H, as well as all HIV-1 group N, O, and P Vprs, activated NFAT. D_lo, a long version of an HIV-1 M subtype D Vpr (D_sh) that has been shown to be inactive in inducing G2 arrest (44), was unable to activate NFAT (Fig. 1A). As a downstream effect of NFAT induction, T cell activation can be assessed by measuring the surface expression of CD69, an early marker of T cell activation. Consistent with their ability to activate NFAT, all tested Vprs, except HIV-1 M D_lo, increased CD69 levels (Fig. 1B). Moreover, Vpr-mediated NFAT activation and cell surface CD69 levels showed a highly significant correlation (Fig. 1C; *R*^2^ = 0.79, *P* = 0.003), supporting our previous finding that Vpr-mediated NFAT activity and CD4+ T cell activation are functionally interconnected (21). As it is still controversial to what extent lentiviral Vprs modulate NF-κB activation (29, 50–52), we used the same lentiviral vectors to transduce Jurkat-dual cells that express luciferase under the control of an NF-κB-dependent promoter. Comparing NF-κB-driven luciferase expression in Vpr-transduced and PHA-treated Jurkat-dual cells to that in cells transduced with the GFP-only expressing lentivirus, we found no evidence for Vpr-mediated activation of NF-κB (Fig. 1D). Thus, at least in the context of this Jurkat T cell reporter system, Vpr boosts the activation of NFAT, but not NF-κB.

### Vpr-induced changes in the transcriptome of HIV-1-infected primary CD4+ T cells

**Fig 2:**
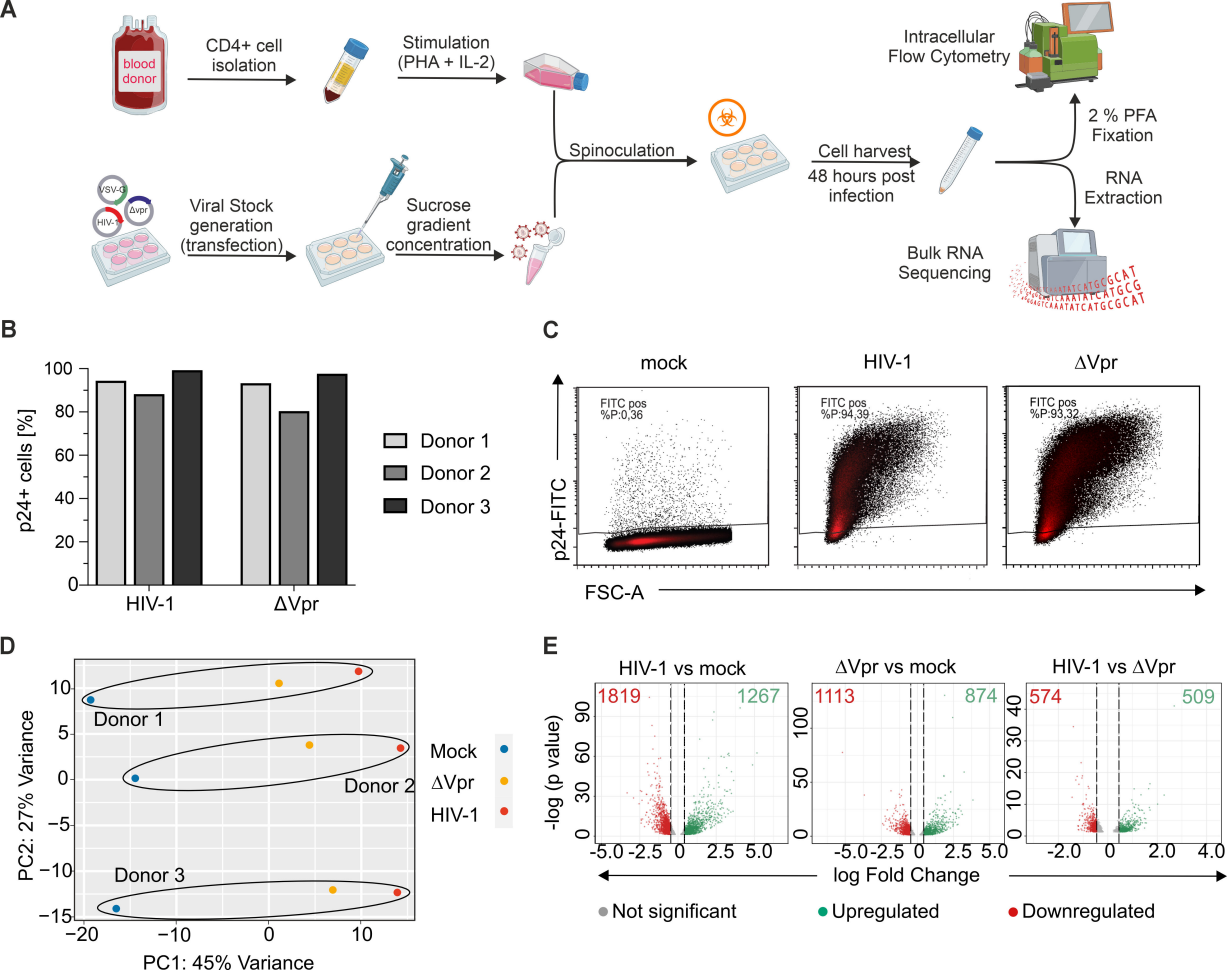
Vpr induces widespread transcriptomic alterations in HIV-1-infected primary CD4^+^ T cells. (**A**) Experimental setup. Pre-stimulated primary CD4^+^ T cells were infected with highly concentrated VSV-G-pseudotyped HIV-1 (wild type or HIV-1 △*vpr*). CD4^+^ T cells were harvested at 48 h post-infection and underwent RNA extraction for RNA-seq. An aliquot was taken and fixed with 2% PFA for flow cytometric analysis. RNA-seq data were analyzed using DESeq2 as described in Materials and Methods. Figure was generated using https://www.biorender.com/. (**B**) Analysis of the p24 capsid-positive population from three different donors and (**C**) gating strategy used to determine the percentage of p24^+^ population at 48 h post-infection. (**D**) Principal component analysis plot of the transcriptomes of mock and HIV-1/△*vpr*-infected CD4^+^ T cells. Three replicates were performed for each infection condition. (**E**) Volcano plots depicting differentially expressed genes between infection conditions after applying the following cutoff criteria: *P*-adj < 0.05; base mean > 100. Red depicts significantly downregulated genes (dots) and the total downregulated gene count (numbers). Green depicts significantly upregulated genes (dots) and the total upregulated gene count (numbers). Gene sets and the corresponding filtering are detailed in Data sets S1 through S3.

Since Vpr stimulates NFAT activity, it is tempting to speculate that it also deregulates the transcriptome in HIV-1-infected cells to a profile that resembles NFAT-induced transcription. However, Vpr is a multifunctional protein that also directly changes the proteome of infected cells, potentially affecting many transcriptional networks (12). Hence, it has remained largely unclear if Vpr-associated transcriptional changes are driven by a broad range of factors or can be attributed primarily to the altered activity of a selected group of key transcription factors. To address this point, we first established a high-efficiency HIV-1 infection protocol allowing infection rates in primary CD4+ T cells >80% (Fig. 2A). For this, pre-activated CD4+ T cells were infected with purified and concentrated HIV-1 stocks via spinoculation. Forty-eight hours later, infection rates were determined by flow cytometry (Fig. 2B and C), and CD4+ T cells of three healthy donors were subjected to RNA extraction and RNA-seq. Principal component analysis revealed consistent clusters based on the infection status of the cells, i.e., mock, HIV-1, or HIV-1 Δ*vpr,* and additional sample distance due to donor variations (Fig. 2D). While wild-type HIV-1 infection has a pronounced effect on the transcriptome of CD4+ T cells, a marked fraction of these changes is indeed mediated by Vpr (Fig. 2D).

In numbers, after filtering for significant genes with a *q* < 0.05 according to Benjamini-Hochberg FDR correction, wild-type HIV-1 infection caused differential expression of 5,859 out of 25,894 genes analyzed (HIV-1 vs mock, Data set S1). Gene set enrichment analyses revealed that HIV-1 infection activated pathways involved in immune activation, increased cytokine signaling, and innate immune response, whereas pathways associated with cell cycle control and DNA replication were downregulated (Fig. S1). This is in line with earlier studies analyzing transcriptomic changes upon HIV-1 infection in primary CD4+ T cells and T cell lines (51, 53). For further analyses, we filtered our hits according to a base mean count > 100 to address only those that are robustly expressed in CD4+ T cells and set the threshold for the log2 fold change to > 0.45 or < −0.45. This resulted in a final set of 3,086 deregulated genes upon HIV-1 infection (Fig. 2E, HIV-1 vs mock; Data set S1), 1,987 genes that were differentially regulated when HIV-1 Δ*vpr* was compared to mock (Data set S2), as well as 509 genes upregulated and 574 genes downregulated in a Vpr-dependent manner (Fig. 2E, HIV-1 wild type vs Δ*vpr*; Data set S3). Altogether, our data show that HIV-1 induces massive changes in the transcriptome of virally infected cells, and approximately one-third of these genes are modulated in a Vpr-dependent manner.

### Vpr deregulates NFAT-controlled genes, induces activatory immune cell signatures, and downregulates gene clusters involved in cell cycle control

**Fig 3:**
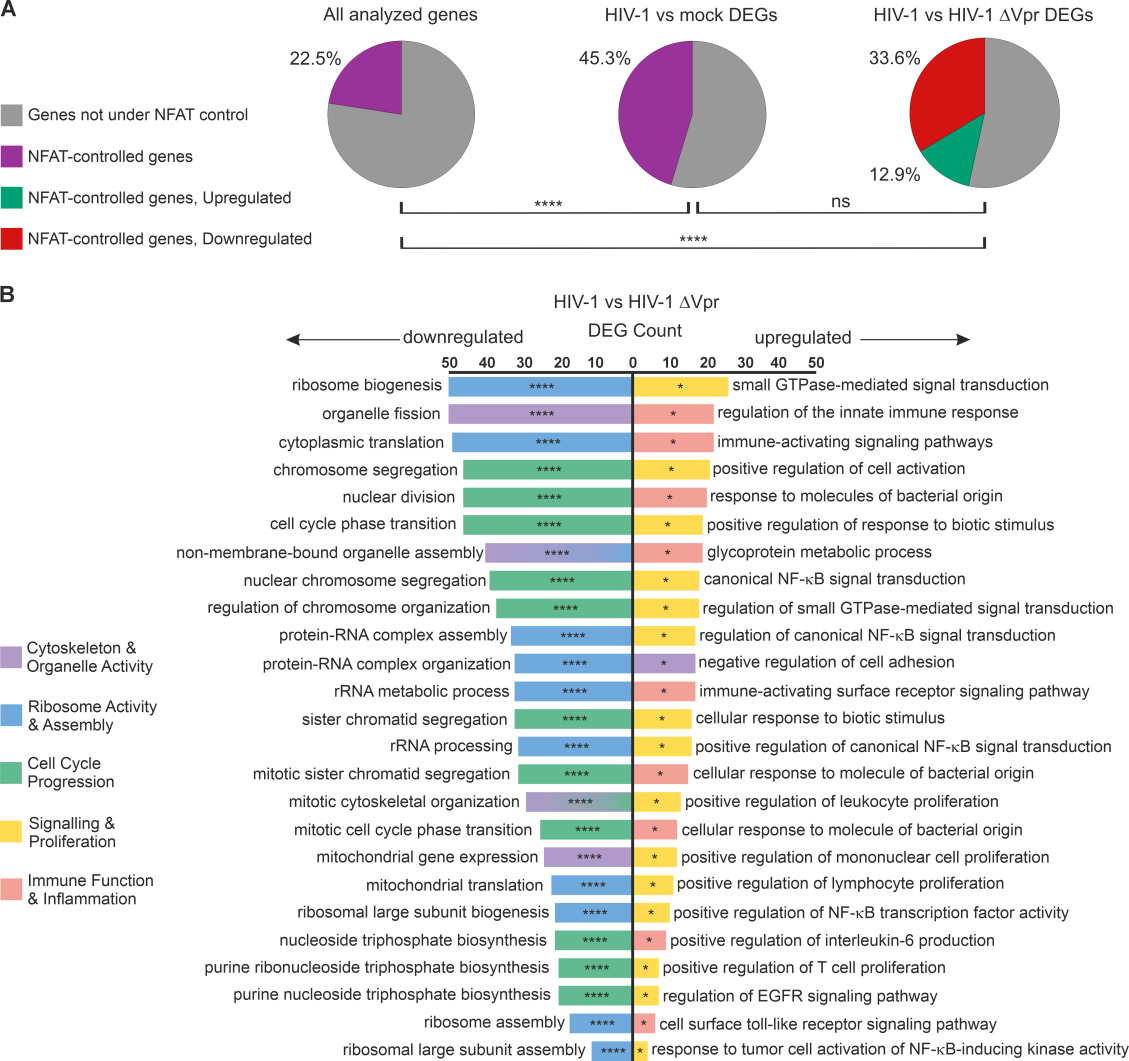
Vpr dysregulates NFAT-controlled genes involved in multiple biological processes. (**A**) Pie charts illustrating the percentage of NFAT-controlled genes among different gene sets, based on a data set of NFATc1-controlled genes extracted from the Harmonizome 3.0 database. Statistical significance was calculated using two-sided Fisher’s exact tests independently for each comparison; ****P < 0.0001. (**B**) Tornado plot depicting the top 25 up- and downregulated biological processes (GO terms) upon infection with HIV-1. Vpr suppresses ribosomal activity and assembly (blue), cell cycle progression (green), and cytoskeleton/organelle activity (purple), while upregulating signaling and proliferation (yellow) along with immune function and inflammation (red). This analysis was performed using EnrichGO, employing the DESeq2-processed RNA-seq results. The hit list was subjected to the following cutoff criteria: adjusted *P*-value < 0.05, base mean > 100, log2 fold change > 0.45 or < −0.45. *q < 0.1; **q < 0.01; ***q < 0.001; and ****q < 0.0001. The Harmonizome-derived gene list referring to NFAT-controlled genes and the downstream processing is detailed in Data set S4.

We next investigated whether there is an enrichment of NFAT-controlled genes in the Vpr-deregulated transcriptome of primary CD4+ T cells. From the five NFAT family member genes, NFATc1 is considered the most important in primary CD4+ T cells (39). Therefore, we utilized a list of NFATc1-controlled genes extracted from the Harmonizome 3.0 database (54) comprising 6,812 transcripts (https://maayanlab.cloud/Harmonizome/gene_set/NFATC1/ENCODE+Transcription+Factor+Targets). Among the 25,840 genes analyzed in our RNA-seq approach, 5,840 (22.5%) are regulated by NFAT (Fig. 3A). Of the 3,086 genes that are differentially expressed upon HIV-1 wild-type infection (Fig. 2E, left panel), 45.3% are controlled by NFAT. Finally, when specifically filtering for genes that are deregulated by HIV-1 Vpr, 504 genes out of 1,083 (46.5%) are NFAT-controlled (Fig. 3A). Hence, nearly half of the transcriptomic changes induced by Vpr are associated with NFAT (see Data set S4 for details).

To functionally address the relevance of Vpr-deregulated genes, we performed comprehensive gene ontology analysis (Fig. 3B). Consistent with a previous study focusing on early Vpr-induced transcriptomic changes (53), gene clusters involved in immune cell activation, signaling, and inflammation were among the top 25 biological processes upregulated by Vpr. Surprisingly, biological processes that were negatively regulated by Vpr were exclusively related to gene clusters mediating organization of the cytoskeleton, ribosomal activity, and genes controlling cell cycle progression (Fig. 3B).

These findings support the hypothesis that HIV-1 Vpr promotes a transcriptional signature in T cells resembling NFAT and T cell activation, which primes infected T cells for productive infection (21). Notably, Vpr also downregulates gene signatures that could be involved in Vpr-mediated G2 arrest.

### Vpr induces transcriptional changes in specific genes involved in immune signaling and cell cycle control

**Fig 4:**
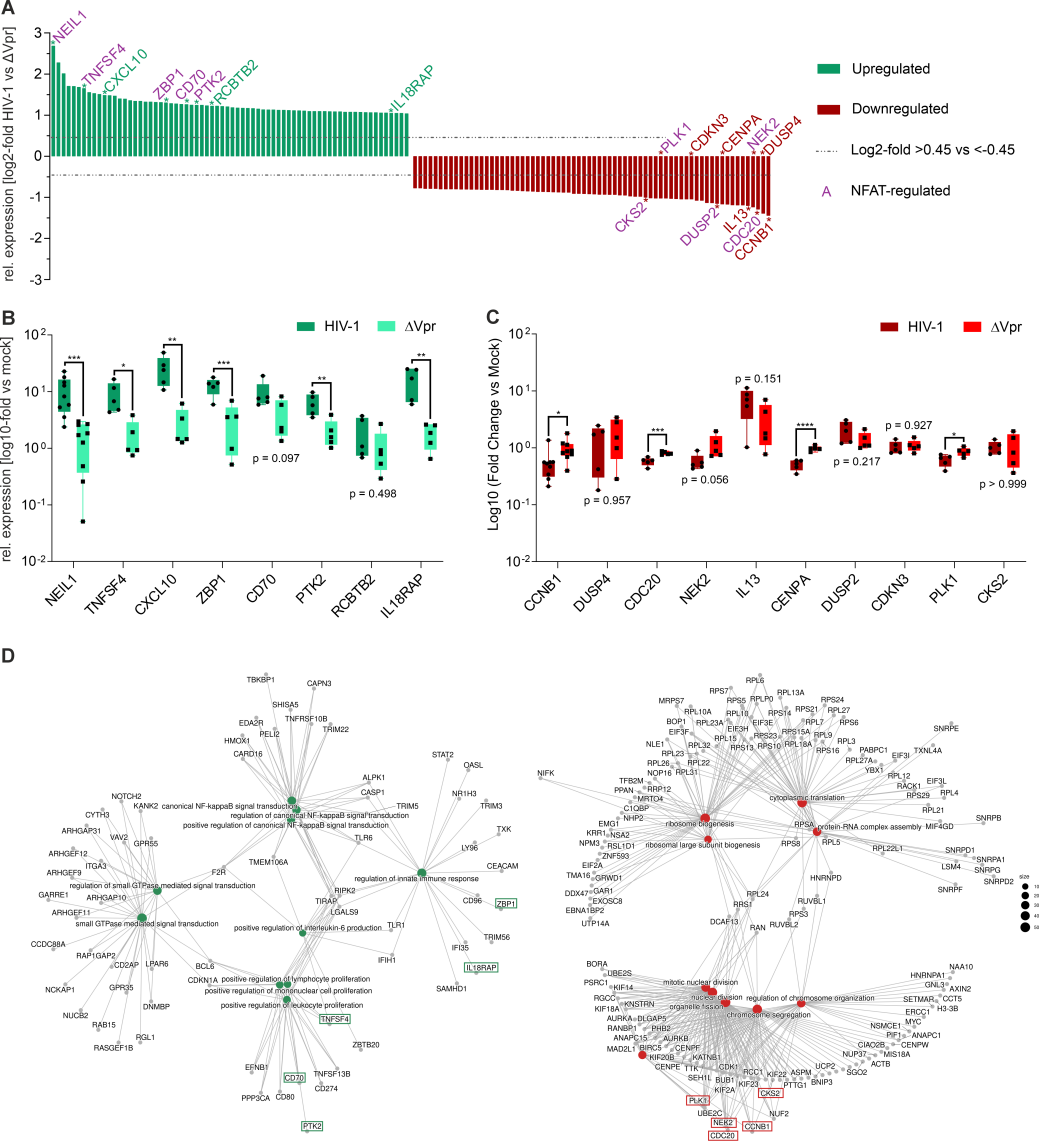
Vpr influences various key biological processes at the RNA level in stimulated CD4^+^ T cells. (**A**) Waterfall plot illustrating the top 70 genes significantly up- or downregulated by Vpr, ordered by log2 FC. Genes selected for validation via qPCR are marked with asterisks and labeled accordingly. NFAT-controlled genes are highlighted in purple, irrespective of their dysregulation status. qPCR analysis of selected upregulated (**B**) and downregulated (**C**) genes. Gene expression (normalized to mock) derived from the geometric mean of three technical replicate Cp values is shown; *n* = 5 (mean with whiskers span min-max). Statistical significance for each gene hit was calculated using a one-way ANOVA coupled to Šidák’s multiple comparisons test using donor-matched (paired) comparisons. (normalized to mock). α = 0.05; *P < 0.05; **P < 0.01; ***P < 0.001; and ****P < 0.0001. (**D**) CNET plots depicting the top 10 up- (left, green) and downregulated (red, right) biological processes as a network in the HIV-1 wt vs HIV-1 △*vpr* comparison. This analysis was performed using EnrichGO, employing the DESeq2-processed RNA-seq results partly depicted in Fig. 2D through E as input. The hit list was subjected to the following cutoff criteria: adjusted *P*-value < 0.05, base mean > 100, and log2 fold change > 0.45 or < −0.45. Gene hits analyzed by qPCR are highlighted in red (downregulated) or green (upregulated).

To validate differential expression of selective candidate genes in the RNA-seq data set, we performed quantitative real-time PCR (qRT-PCR) of CD4+ T cells from independent donors. For validation, we selected genes among the top 70 up- and downregulated hits that are either involved in immune signaling and activation (*TNFSF4*, *CXCL10*, *ZBP1*, *CD70*, *IL18RAP*, *DUSP4*, *IL13*, and *DUSP2*) or cell cycle control (*NEIL1*, *PTK2*, *RCBTB2*, *CCNB1*, *CDC20*, *NEK2*, *CENPA*, *CDKN3*, *PLK1*, and *CKS2*) (Fig. 4A). Out of the 18 selected genes, 10 genes are NFATc1-controlled (Fig. 4A, genes labeled in purple). qPCR validated Vpr-dependent upregulation of all eight candidate genes selected, although two (*RCBTB2* and *CD70*) failed to reach statistical significance (Fig. 4B). Furthermore, we verified the Vpr-driven downregulation of *CCNB1*, *CDC20*, *CENPA,* and *PLK1* (Fig. 4C). In order to link and visualize the specific Vpr-deregulated genes (Fig. 2E) into biological networks, we generated CNET plots of the top 10 upregulated and downregulated biological processes (GO terms from EnrichGO, Fig. 3). These highlight the differential clusters of pathways that are triggered by Vpr-upregulated genes, i.e., stimulation of lymphocyte/leukocyte activation and the innate immune response as well as NF-κB signaling (Fig. 4D, left). Biological processes affected by Vpr-downregulated genes can be grouped into two large clusters: (i) ribosomal assembly and translation, (ii) cell cycle control, nuclear division, and chromosome organization (Fig. 4D). All genes verified to be downregulated by Vpr (*CCNB1*, *CDC20*, *NEK2*, and *PLK1*) represent major nodes in the biological networks represented by the CNET plots for mitotic nuclear division, nuclear division, chromosome segregation, and regulation of chromosome organization. Conversely, the Vpr-induced genes *CD70*, *PTK2,* and *TNFSF4* are involved in positive regulation of lymphocyte/leukocyte proliferation*,* whereas *ZBP1* and *IL18RAP* are associated with regulation of innate immune response.

In summary, specific genes upregulated by Vpr and NFAT enhance T cell activation and proliferation and activate innate sensing and inflammation. Meanwhile, Vpr-downregulated genes, which are partly also affected by NFAT, are major players in cell cycle control, nuclear division, and chromosome organization.

### Absence of detectable Vpr-mediated G2 arrest in primary CD4+ T cells

Our gene set enrichment analyses revealed that Vpr downregulates transcriptomic signatures related to ribosome assembly, cytoskeleton organization, and cell cycle regulation (Fig. 3B). This suggests that deregulation of cellular transcripts might contribute to the phenomenon of Vpr-mediated G2 arrest.

**Fig 5:**
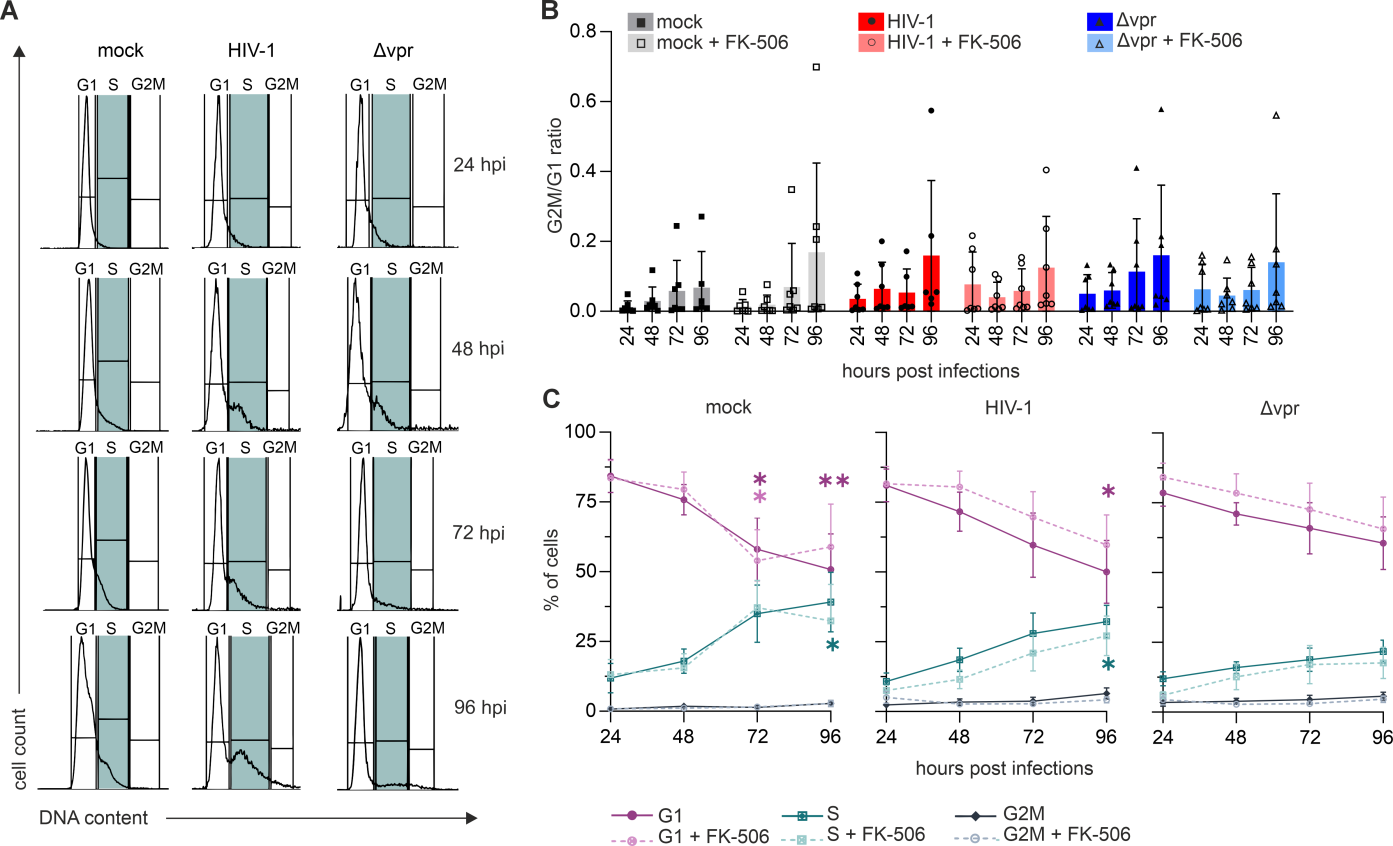
Vpr does not induce G2 arrest in activated primary CD4^+^ T cells. (**A**) Primary CD4^+^ T cells were TCR-stimulated and then mock infected or infected with HIV-1 wt or HIV-1 Δ*vpr*. Cells were analyzed 24–96 h post-infection for their cell cycle phase by PI staining. Representative PI histograms show mock samples gated on singlets and infected samples gated on p24^+^ events; gates delineate G1, S, and G2/M, with S-phase highlighted in green. (**B**) G2M/G1 ratios for TCR-stimulated primary CD4^+^ T cells (mock on singlets; infected on p24^+^) in the presence or absence of FK-506 (10 ng/mL); *n* = 8 (mean ± SD). (**C**) Percent distribution across G1, S, and G2/M for TCR-stimulated primary CD4^+^ T cells in the presence or absence of FK-506 (10 ng/mL); *n* = 8 (mean ± SEM). Statistical analyses were performed using paired two-way ANOVA with Dunnett’s multiple comparisons (α = 0.05; *P < 0.05; **P < 0.01).

We first analyzed G2 cell cycle arrest in HIV-1-infected primary CD4+ T cells, i.e., the model used for our RNA-seq approach (Fig. 5A). CD3/CD28 pre-stimulated CD4+ T cells were infected with equal p24 amounts of HIV-1 or HIV-1 Δ*vpr*. To specifically monitor the cell cycle in HIV-1-infected cells, we performed co-staining of intracellular p24 and nuclear DNA with PI, followed by subsequent flow cytometric analysis. Unexpectedly, we did not observe Vpr-dependent induction of G2 arrest in HIV-1-infected primary CD4+ T cells of any of the donors tested (Fig. 5A and B), irrespective of NFAT activity, although NFAT1, 2, and 4 were clearly detectable in primary CD4+ T cells (Fig. S2). Furthermore, there was no G2 arrest induced by Vpr, with TCR pre-stimulation or not (Fig. S2). Notably, at 72 and 96 hpi, we witnessed a higher proportion of cells in the S phase in mock and HIV-1 infections, whereas the fraction of cells in S remained low in HIV-1 Δ*vpr*-infected primary CD4+ T cells (Fig. 5A). Data from various donors indicate that mock and HIV-1-infected primary T cells progress upon stimulation from G1 into S phase, and in HIV-1-infected cells, this progression can be slowed down by NFAT inhibition (Fig. 5C). Similar to NFAT inhibition in HIV-1 wt-infected cells, fewer cells progressed from G1 into S phase in the absence of functional Vpr expression (Fig. 5C).

### NFAT activation is required for Vpr-induced G2 cell cycle arrest in Jurkat T cells

**Fig 6:**
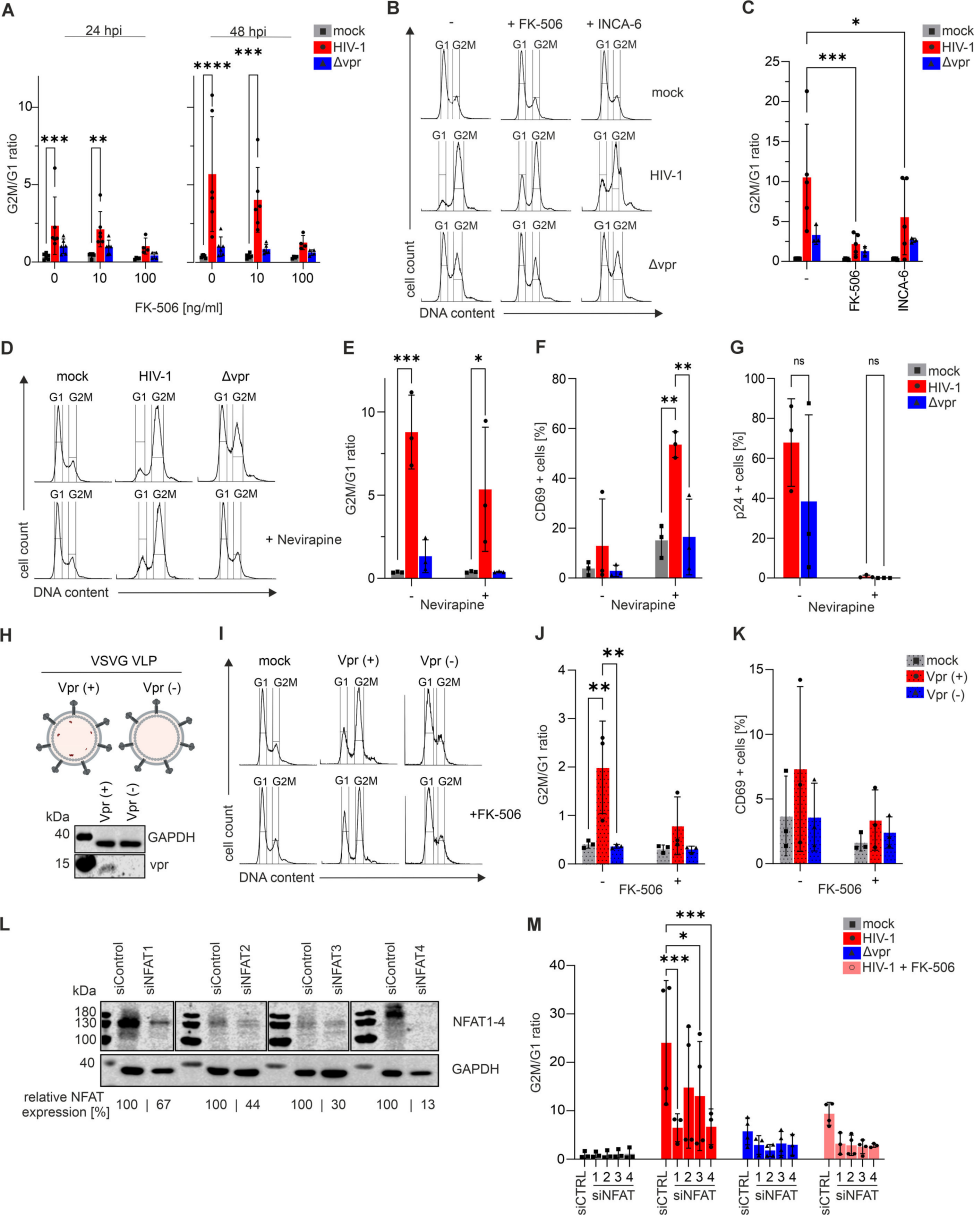
Vpr-mediated G2 arrest in Jurkat cells is NFAT dependent. (**A**) Jurkat E6.1 cells were infected with HIV-1 wt or HIV-1 Δ*vpr* in the presence or absence of FK-506 (10 or 100 ng/mL). G2M/G1 ratios were calculated (mock on singlets; infected on p24^+^); *n* = 4–6 (mean ± SD). (**B**) Representative PI histograms show infected Jurkat E6.1 cells in the presence or absence of 10 ng/mL FK-506 or 2.5 µM INCA-6 (mock singlets; infected p24^+^); gates delineate G1 and G2/M. (**C**) Cell cycle arrest of infected and NFAT-inhibited Jurkat E6.1 cells was evaluated by calculating the G2M/G1 ratio *n* = 3–5 (mean ± SD). (**D**) Jurkat E6.1 cells were infected with HIV-1 or HIV-1 Δ*vpr* in the presence or absence of nevirapine (10 µM). Representative PI histograms show cell cycle distribution; gates delineate G1 and G2/M. (**E**) Cell cycle arrest of infected and nevirapine-treated Jurkat E6.1 cells was evaluated by calculating the G2M/G1 ratio; *n* = 3 (mean ± SD). (**F**) Percentage of CD69^+^ and (**G**) p24^+^ cells was evaluated in infected and nevirapine-treated Jurkat E6.1 cells; *n* = 3 (mean ± SD). (**H**) Scheme of VLPs used in panels I–K and immunoblot of VLP-transduced Jurkat E6.1 cells confirming Vpr delivery following transduction with Vpr-containing VLPs. (**I**) Representative PI histograms of VLP-transduced Jurkat cells in the presence or absence of FK-506 (100 ng/mL); gates delineate G1 and G2/M. (**J**) Cell cycle arrest was assessed by calculating the G2M/G1 ratio of VLP-transduced Jurkat E6.1 cells; *n* = 3 (mean ± SD). (**K**) Percentage of CD69^+^ VLP-transduced Jurkat cells; *n* = 3 (mean ± SD). (**L**) siRNA-mediated knockdown of NFAT1–4 in Jurkat E6.1 cells was confirmed by immunoblotting 4 days post-electroporation, and the representative immunoblot of the individual knockdowns is shown. NFAT signals were normalized to GAPDH and calculated relative to siCTRL. (**M**) Two days post-electroporation, NFAT-knockdown cells were infected with HIV-1 in the presence or absence of FK-506 (100 ng/mL). Cell cycle arrest was evaluated by G2M/G1 ratio (mock on singlets; infected on p24^+^); *n* = 3–4 (mean ± SD). Statistical significance was calculated using ordinary two-way ANOVA with Dunnett’s or Šídák’s multiple comparisons (α = 0.05, *P < 0.05; **P < 0.01; ***P < 0.001; and ****P < 0.001).

Vpr-mediated G2 arrest is often monitored in lymphoma-derived immortalized T cell lines, e.g., Jurkat cells (21, 22, 55). Therefore, we repeated the experiment in Jurkat T cells and found, in agreement with previous data, Vpr-dependent G2 arrest in HIV-1-infected cells at 24 hpi. This effect was even more pronounced at 48 hpi (Fig. 6A and B). Vpr also augments NFAT activation in Jurkat T cells (Fig. 1), and our previous findings revealed a correlation between the impaired capability of Vpr mutants to activate NFAT and their inability to induce G2 arrest (21). We hence hypothesized that NFAT activation by Vpr is an upstream regulatory event involved in G2 arrest in Jurkat T cells.

To test this, Jurkat T cells were infected with HIV-1 or HIV-1 Δ*vpr* in the presence or absence of the NFAT inhibitor FK-506. Inhibitor treatment of the immortalized Jurkat T cells did not alter infection rates that were reproducibly high in this system (Fig. S3A), whereas T cell activation was blocked by FK-506 as evident from reduced CD69 levels (Fig. S3B). Infected cells were identified via intracellular p24 staining, and G2 arrest was quantified via PI staining and subsequent flow cytometry (Fig. 6A and B). Remarkably, NFAT inhibition by FK-506 in HIV-1-infected Jurkat T cells clearly prevented Vpr from inducing a G2 cell cycle arrest, and a similar effect was measured upon utilization of the specific NFAT inhibitor INCA-6 (Fig. 6B and C), which likewise did not affect infection rates in this setting and had only a minor impact on CD69 levels (Fig. S3C and D). To verify that incoming, i.e., virion-associated, Vpr is sufficient to induce G2 arrest independently of productive infection, we blocked reverse transcription by utilizing the RT-inhibitor nevirapine (Fig. 6D through F). In this setting, when infecting Jurkat cells with equal p24 amounts of WT or Δ*vpr* HIV-1, Vpr triggered a G2 cell cycle arrest (Fig. 6D and E) and induced CD69 (Fig. 6F) in the absence of productive infection (Fig. 6G). As an alternative approach, we employed VLPs carrying Vpr (Fig. 6H). Only VLPs loaded with Vpr induced a G2 arrest (Fig. 6I and J) and triggered early T cell activation as measured by CD69 expression (Fig. 6K). This phenotype could be reverted by blocking NFAT with FK-506 (Fig. 6J and K). To unambiguously verify that Vpr-induced G2 arrest in Jurkat T cells is mediated by NFAT, we knocked down the individual NFAT proteins by siRNA (Fig. 6L), which did not affect HIV-1 infection rates in general (Fig. S6E). Of note, knockdown of NFAT1 or NFAT4, which are strongly expressed in Jurkat T cells (Fig. 6L), markedly reduced the ability of Vpr to induce G2 arrest, similar to inhibition of NFAT by FK-506 (Fig. 6M).

Taken together, Vpr does not seem to induce G2 arrest in primary CD4+ T cells, whereas it is able to do so in the immortalized Jurkat T cell line. This Jurkat T cell phenomenon is NFAT dependent, which agrees with the deregulation of gene transcripts by Vpr seen in primary CD4+ T cells.

### Inhibition of NFAT abrogates Vpr-mediated enhancement of HIV-1 infection and p24 production in primary CD4+ T cells

**Fig 7:**
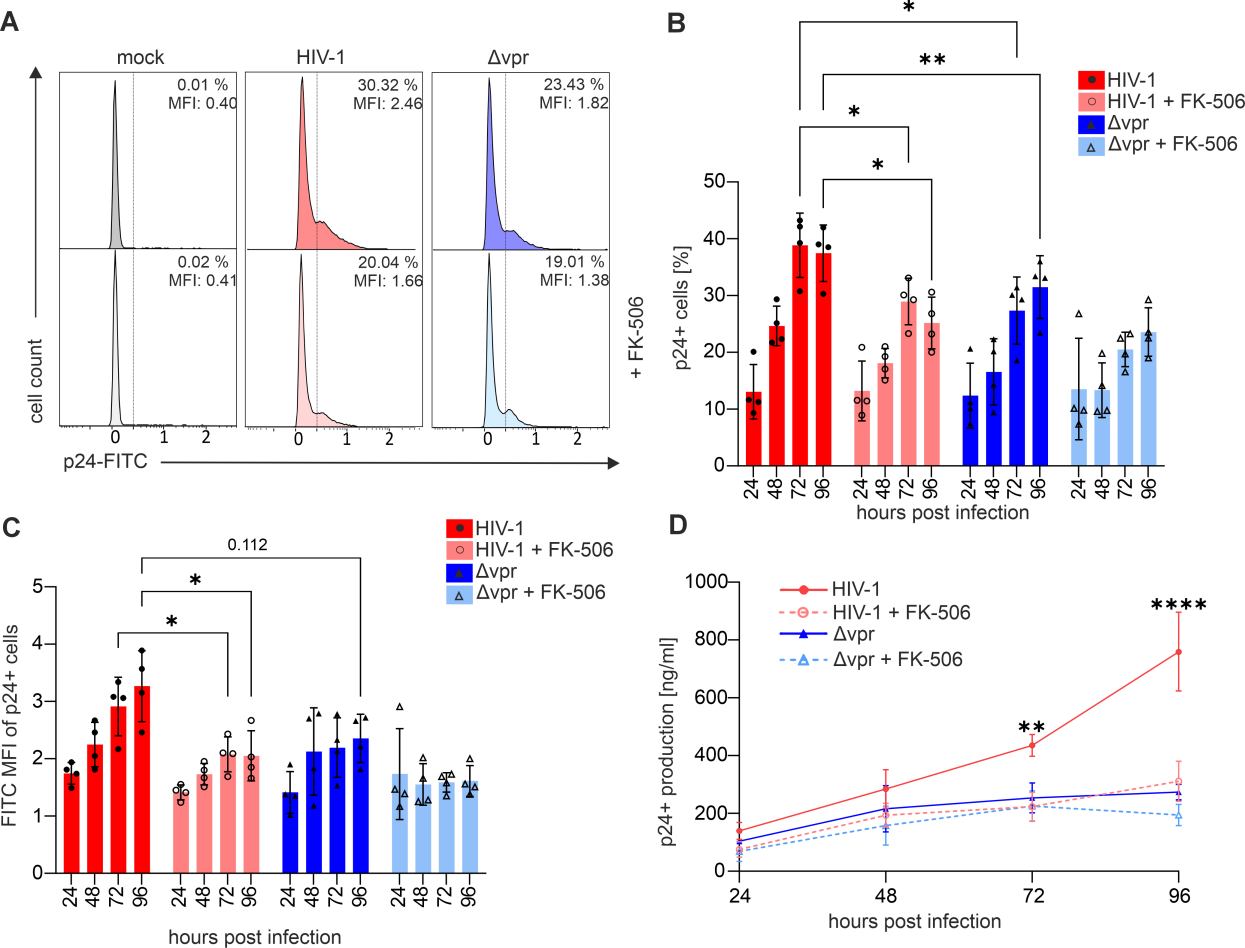
Inhibition of NFAT abrogates Vpr-mediated enhancement of HIV-1 infection and p24 production in primary CD4^+^ T cells. (**A**) Representative histograms illustrating p24^+^ expression in TCR-stimulated HIV-1 wt or HIV-1 Δ*vpr*-infected CD4^+^ T cells at 96 h post-infection in the presence or absence of FK-506 (10 ng/mL), indicated by the percentage of p24-positive cells and the MFI of the p24 signal. (**B**) Infection rates of TCR-stimulated HIV-1 wt or HIV-1 Δ*vpr*-infected CD4^+^ T cells in the presence or absence of FK-506 (10 ng/mL) at various time points post-infection; *n* = 4 (mean ± SD) and (**C**) respective MFI; *n* = 4 (mean ± SD). (**D**) Supernatant p24 titers of TCR-stimulated HIV-1-infected CD4^+^ T cells in the presence or absence of FK-506 (10 ng/mL) at various time points post-infection; *n* = 4 (mean ± SD). Statistical analyses were performed using paired two-way ANOVA with Šídák’s multiple comparisons test (α = 0.05; *P < 0.05; **P < 0.01; and ****P < 0.0001).

Activation of NFAT-controlled pathways that stimulate T cell activation (Fig. 3) might support and enhance HIV-1 replication. Therefore, to define the importance of NFAT in Vpr-mediated enhancement of HIV-1 infection, we monitored HIV-1 replication in CD4+ T cells upon NFAT inhibition. To this end, CD3/CD28-stimulated primary CD4+ T cells were infected with equal p24 amounts of HIV-1 expressing an intact or defective *vpr*-ORF, and replication was monitored in the presence or absence of the NFAT inhibitor FK-506 over a period of 4 days (Fig. 7A through D). Samples of the infected cell cultures were taken in 1-day intervals, and the frequency of infected cells was quantified by intracellular p24 staining (Fig. 7A). Flow cytometry revealed that Vpr enhanced infection levels by around one-third with significant differences becoming obvious at 72 and 96 h post-infection (Fig. 7B). Importantly, NFAT inhibition with FK-506 reduced the number of infected cells in the cultures and abrogated Vpr-mediated enhancement of infection, indicating that this phenomenon is indeed NFAT dependent (Fig. 7B). Notably, Vpr-mediated enhancement of infection was not only measurable when analyzing the total number of infected, i.e., p24+ cells, but also when calculating the p24-FITC mean fluorescence intensity (MFI), as a proxy of HIV-1 production per cell (Fig. 7C). Moreover, we observed a three to fourfold reduced capacity of HIV-1 Δ*vpr*-infected CD4+ T cells to release p24 into cell culture supernatants at 96 hpi (Fig. 7D), and this was also true when cells were pre-stimulated with PHA instead of CD3/CD28 (Fig. S4). Again, this increase in p24 production was reduced to Δ*vpr* levels when NFAT was blocked by FK-506 (Fig. 7D). In conclusion, Vpr augments HIV-1 replication in primary CD4+ T cells in an NFAT-dependent manner.

## DISCUSSION

Within this study, we provide new insights into the role of Vpr in activating NFAT and how this, in turn, contributes to the profound transcriptomic changes induced by HIV-1 in general and Vpr in particular. We report functional conservation of Vpr-mediated NFAT activation across HIV-1 groups and subtypes. Moreover, we demonstrate that a substantial fraction of Vpr-induced transcriptional changes is attributable to dysregulated NFAT activity, which is remarkable given the plethora of Vpr-induced proteasomal and epigenetic dysregulations (12, 43, 56). Importantly, all of these experiments were performed in primary CD4+ T cells, demonstrating that Vpr is indeed able to augment HIV-1 replication and virus production in a Vpr- and NFAT-dependent manner in this key HIV-1 target cell type, in addition to macrophages (57, 58). It is evident that this mechanism plays a crucial role in boosting HIV-1 replication, which heavily relies on an activated T cell environment for productive infection. However, the precise mechanism by which Vpr activates NFAT remains unclear. Virion-delivered Vpr has been reported to induce Ca²^+^ influx in target cells, and in Jurkat T cells, Vpr can further potentiate Nef-driven NFAT activation downstream of proximal Ca²^+^ signaling (21). Furthermore, in resting CD4^+^ T cells and primary macrophages, Vpr enhanced NFAT nuclear translocation, and inhibition of the endogenous NFAT-export kinase GSK3β was sufficient to restore NFAT nuclear accumulation in the absence of Vpr, suggesting that Vpr may interfere with GSK3β-mediated NFAT export (21). Although we did not dissect these upstream events in primary CD4^+^ T cells, our transcriptomic data demonstrate that Vpr activates NFAT and thereby drives an NFAT-controlled transcriptional reprogramming in CD4^+^ T cells.

Furthermore, our results reveal a surprising role of NFAT in the long-recognized phenomenon of Vpr-mediated G2 cell cycle arrest in Jurkat T cells. First, pathways and genes downregulated by Vpr are enriched in genes involved in regulating the cell cycle and associated processes. Second, inhibition of NFAT by the well-established drug FK506 and the NFAT inhibitor INCA6 blocked Vpr-mediated G2 arrest in HIV-1-infected Jurkat T cells, similar to siRNA-mediated knockdown of NFAT, clearly indicating that NFAT is required for this process in immortalized T cells and acts upstream of the cell cycle block. NFAT’s role in this was previously suggested by work showing that the ability of Vpr mutants to activate NFAT, induce G2 arrest, and transactivate the HIV-1 LTR in Jurkat T cells strongly correlates with each other (21). The latter is compelling given the presence of NFAT binding sites within the HIV-1 LTR promoter (59, 60). However, how this observation aligns with the overwhelming body of data interconnecting Vpr’s ability to degrade host cell factors via the DCAF1-CUL4 E3 ligase complex with G2 arrest is currently unclear. Members of the NFAT transcription factor family are known to regulate cell cycle progression, with different family members exerting context-dependent effects (61). Thus, NFAT activation could indeed govern G2 arrest, for instance, via negative modulation of *CCNB1* through NFAT-mediated induction of p21 (61). In line with this, Vpr mutants that are unable to activate NFAT also fail to induce G2 arrest in Jurkat T cells (21), further supporting a functional link between NFAT activation and Vpr-mediated cell cycle control. Consistent with such a mechanism, *CCNB1* was also found to be downregulated by Vpr in our RNA-seq data and validated by qRT-PCR (Fig. 4).

Moreover, it is important to emphasize that we were unable to detect a Vpr-mediated G2 arrest in stimulated and resting primary CD4+ T cells, while it was clearly detectable in the immortalized Jurkat T cell line. Although research over the last decades extensively focused on the G2 arrest induced by Vpr, there is, to the best of our knowledge, only sparse evidence for this phenomenon in *ex vivo* cultured and HIV-1-infected primary CD4+ T cells, with one study employing highly stimulated and positively selected CD4+ T cells (30). Hence, the unambiguous confirmation of this phenotype in HIV-1-relevant primary cells is still lacking (29). More specifically, while Vpr has been shown to degrade factors such as MCM10 (62), EXO1 (6), and CCDC137 (24) in cell lines and primary T cells in a DCAF1-dependent manner, the knockdown of these factors often induces cell cycle changes in immortalized cell lines without confirmation in primary T cells. Instead, in HIV-1-infected primary CD4+ T cells, we observed a higher proportion of cells in S in a Vpr-dependent manner, resembling the distribution in mock-infected cells, whereas primary CD4+ T cells infected with Vpr-deficient HIV-1 largely remained in G1 (Fig. 5). Of note, we used HIV-1 with intact Vif, which has also been proposed to arrest cells in G2 (32, 63). This phenotype seems to be species-specific (64) and has predominantly been established in immortalized cell lines that were also used to characterize Vpr-mediated G2 arrest (32–34, 64). Hence, it will be highly interesting and relevant to analyze whether a similar scenario applies for Vif-mediated G2 arrest.

**Fig 8:**
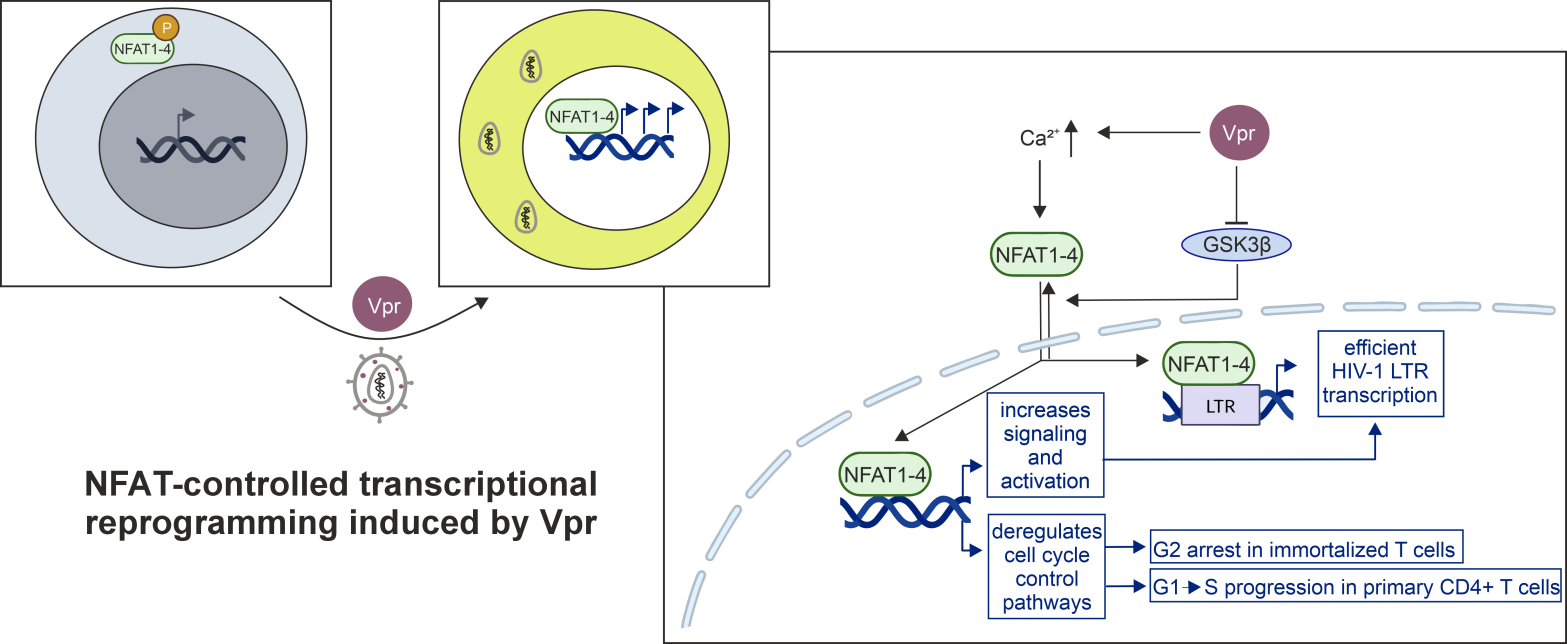
Model of Vpr-mediated NFAT activation. Scheme summarizing the findings of the present study and our previous mechanistic insights into how Vpr might activate NFAT (21). Vpr activates NFAT by increasing cellular Ca^2+^ levels and interference with the NFAT export kinase GSK3β via an as-yet-undefined mechanism. NFAT activation directly enhances HIV-1 LTR transcription. LTR activity is also increased indirectly via NFAT-triggered proinflammatory and activatory signaling cascades. Furthermore, Vpr-mediated NFAT induction deregulates cell cycle control pathways, resulting in G2 arrest in immortalized T cell lines while supporting G1 to S progression in primary CD4^+^ T cells. Figure was generated using https://www.biorender.com/ and CorelDRAW.

Altogether, we propose a model in which Vpr indeed degrades host cell factors involved in DNA-damage repair, cellular homeostasis, and cell cycle control and stimulates NFAT activation in primary CD4+ T cells and Jurkat T cells. In immortalized and artificial cell culture systems, these phenomena result in G2 arrest, either through shared or independent mechanisms. Conversely, in primary T cells, in which early cell cycle arrest would paradoxically result in CD4+ T cell death, the aforementioned phenotypes will optimize reverse transcription and integration of the HIV-1 DNA genome and contribute to efficient LTR transcription in activated and resting CD4+ T cells without a negative impact on T cell proliferation. Of note, recent data, albeit largely based on artificial cell systems, are supportive of such a scenario (65). Overall, our proposed model (Fig. 8) establishes a coherent functional role for Vpr in the early phase of the HIV-1 replication cycle, integrating its ability to manipulate the cell cycle with its activatory effect on viral gene expression.

## Data Availability

All data generated and analyzed during this study are included in this published article. RNA-seq data sets have been deposited in NCBI’s Gene Expression Omnibus (66) and are accessible through GEO Series accession number GSE274705.

## SUPPLEMENTAL MATERIAL

Data set S1 mbio.03605-25-s0001.xlsx List of HIV-1 deregulated genes and the according workflow how the data were processed.

Data set S2 mbio.03605-25-s0002.xlsx List of HIV-1 delVpr vs mock deregulated genes and the according workflow how the data were processed.

Data set S3 mbio.03605-25-s0003.xlsx List of HIV-1 vs delVpr deregulated genes and the according workflow how the data were processed.

Data set S4 mbio.03605-25-s0004.xlsx List of NFATc1 deregulated genes and the overlap to HIV-1 and HIV-1 delVpr deregulated genes.

Supplemental Figures mbio.03605-25-s0005.pdf Figures S1-S4.

ASM does not own the copyrights to Supplemental Material that may be linked to, or accessed through, an article. The authors have granted ASM a non-exclusive, world-wide license to publish the Supplemental Material files. Please contact the corresponding author directly for reuse.
