# Supplementary material for: HIV-1 Vpr induces an NFAT-controlled transcriptional program in primary CD4+ T cells
Source: mBio. 2026 Feb 4;17(3):e03605-25. doi: 10.1128/mbio.03605-25 (PMC12977483; doi:10.1128/mbio.03605-25)
Supplement: Supplemental Figures — Figures S1-S4. [file mbio.03605-25-s0005.pdf]

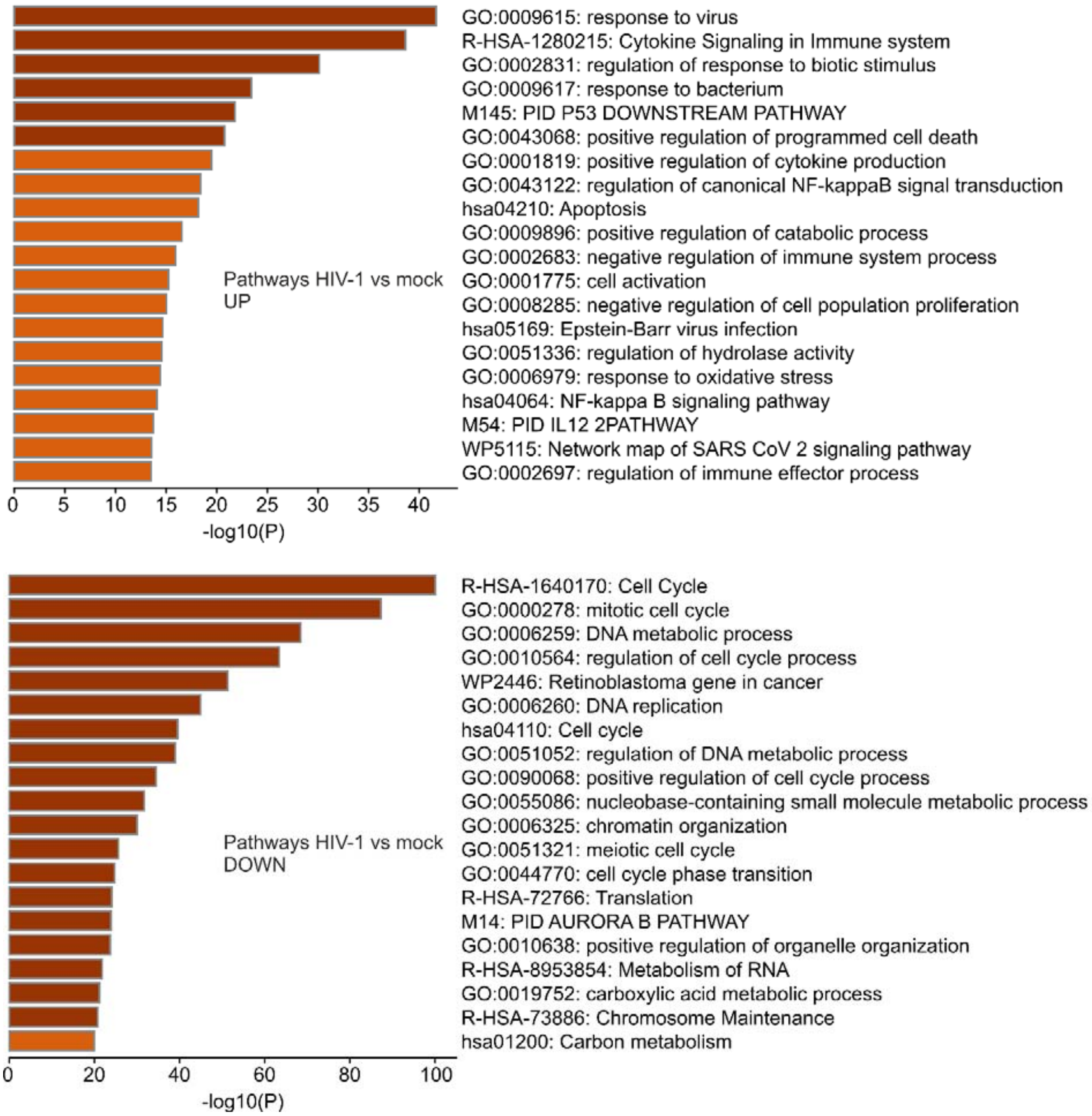


**Supplementary Figure S1. Multi-list meta-analysis of RNA sequencing data.** Metascape bar graphs showing non-redundant enrichment clusters of various HIV-1-dysregulated biological processes. These graphs illustrate the functional clusters most significantly upregulated (top) and downregulated (bottom) within the HIV-1 vs. mock comparative instance. GO term analysis was done based on the gene list detailed in Supplemental Dataset 1.

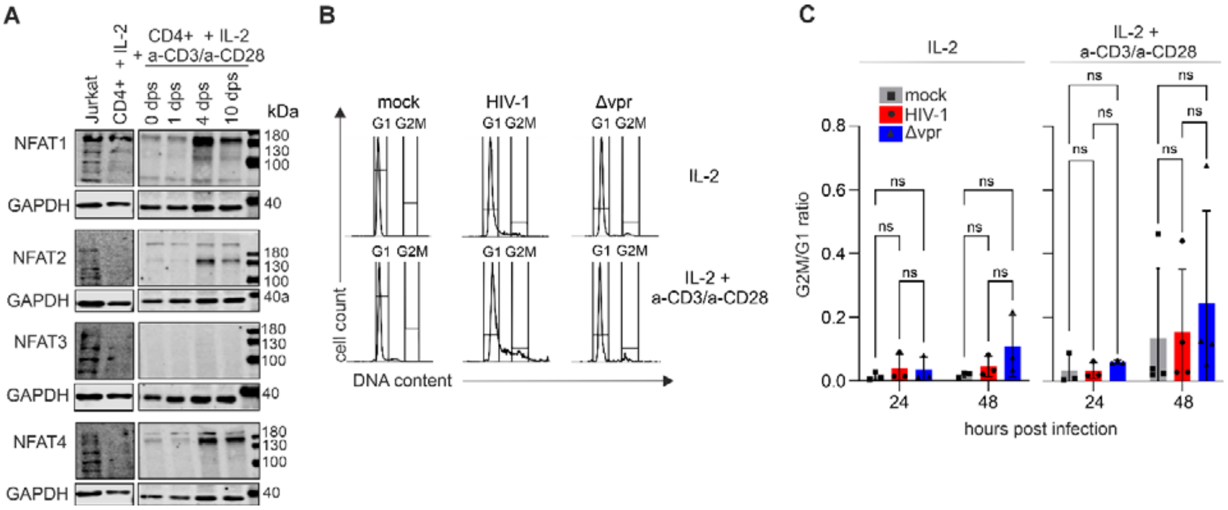


**Supplementary Figure S2. Vpr does not induce G2 arrest in non-activated primary CD4⁺ T cells.** (A) Jurkat E6.1 cells, primary CD4⁺ T cells cultured with IL-2 alone, and TCR-activated primary CD4⁺ T cells (harvested up to 10 days post-stimulation) were analyzed by immunoblot for endogenous NFAT1, NFAT2, NFAT3 and NFAT4 expression; GAPDH was used as loading control. (B) Primary CD4⁺ T cells were cultured with IL-2 alone or were TCR-stimulated one day before infection with HIV-1 wt or HIV-1 *Δvpr*. Representative PI histograms show mock samples gated on singlets and infected samples gated on p24⁺ events; gates delineate G1 and G2/M. (C) Quantification of G2M/G1 ratios for IL-2–treated or TCR-activated primary CD4⁺ T cells (mock singlets; infected p24⁺); n = 3–4 (Mean ± SD). Statistical significance was calculated using ordinary two-way ANOVA with Tukey's multiple comparisons. (α = 0.05, ns = not significant).

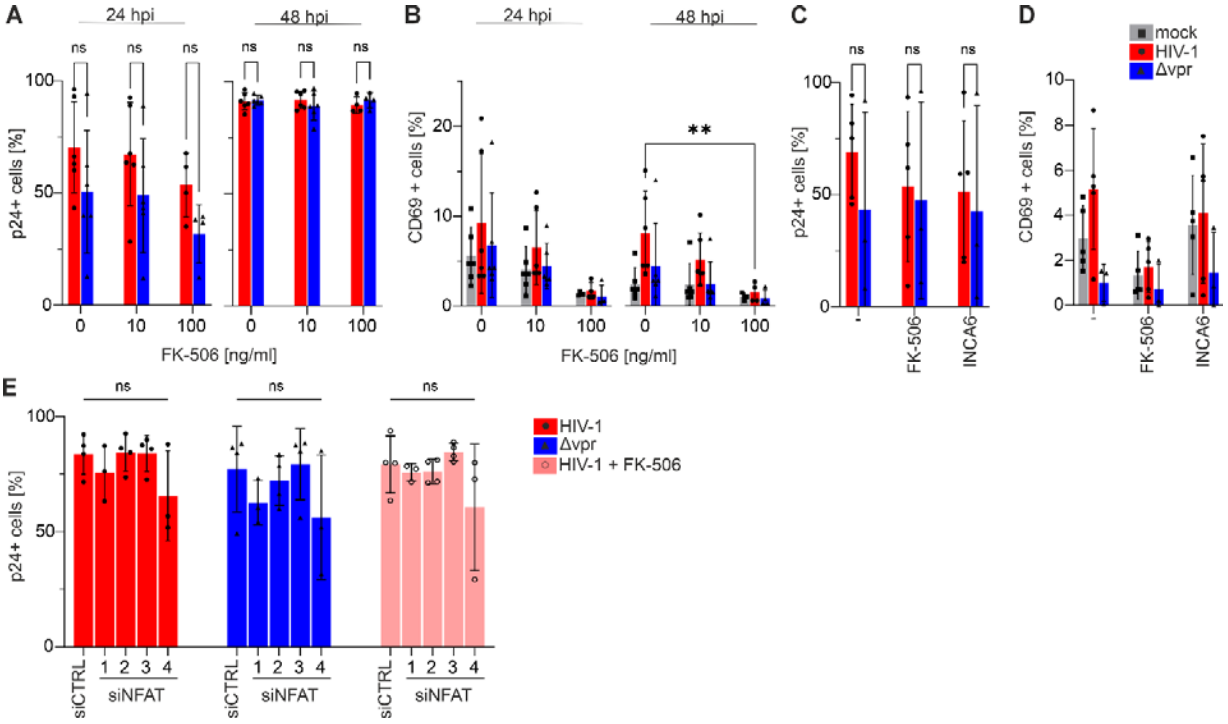


**Supplementary Figure S3. NFAT inhibition does not affect infection rates in Jurkat E6.1 cells and NFAT inhibition reduces CD69 expression.** (A) Jurkat E6.1 cells were infected with HIV-1 , HIV-1 wt or HIV-1 *Δvpr* in the presence or absence of FK-506 (10 or 100 ng/ml). The percentages of p24$^+$ cells and (B) CD69$^+$ cells were quantified by flow cytometry; n = 4–6 (Mean±SD). (C) Jurkat E6.1 cells were infected with HIV-1 in the presence or absence of FK-506 (10 ng/ml) and INCA-6 (2.5 µM) and the percentages of p24$^+$ and (D) CD69$^+$ cells were quantified by flow cytometry; n = 3–5 (Mean±SD). (E) Two days after siRNA-mediated knockdown of NFAT1–4, Jurkat E6.1 cells were infected in the presence or absence of FK-506 (10 ng/ml). The percentages of p24$^+$ cells were determined; n=3-4 (Mean ± SD). Statistical significance was calculated using ordinary two-way ANOVA with Dunnett´s or Šídák's multiple comparisons ($\alpha$ = 0.05, ** p < 0.01; **** p < 0.0001; ns = not significant).

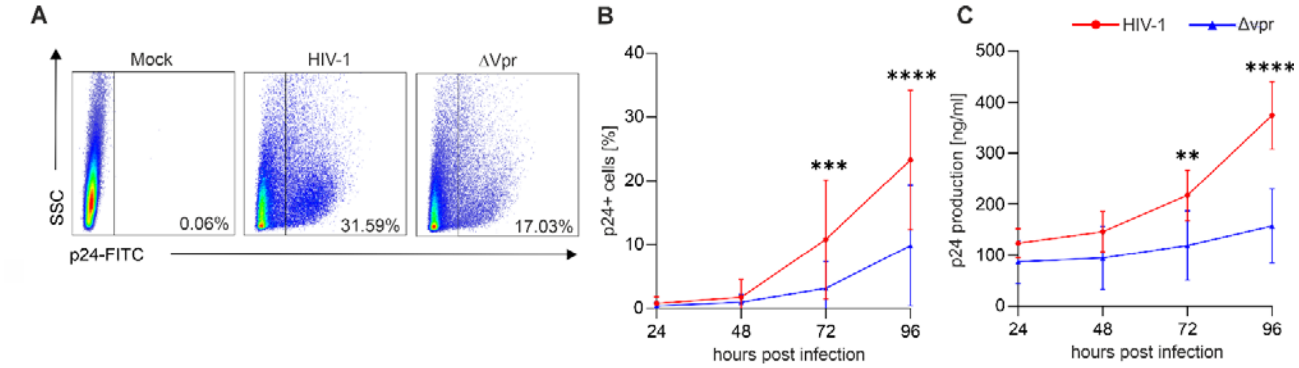


**Supplementary Figure S4. Vpr boosts infection in PHA-stimulated primary CD4$^+$ T cells.** (A) Representative dot plots illustrating p24$^+$ expression in PHA-stimulated HIV-1 wt or HIV-1 *Δvpr* infected CD4$^+$ T cells at 96 hours post-infection, indicated by the percentage of p24-positive cells. (B) Infection rates of PHA-stimulated HIV-1–infected CD4$^+$ T cells at various time points post-infection; n = 12 (Mean ± SD). (C) Supernatant p24 titers of PHA-stimulated, HIV-1–infected CD4$^+$ T cells at various time points post-infection; n = 8 (Mean ± SD). Statistical analyses were performed using paired two-way ANOVA with Šídák's multiple comparisons test (α = 0.05; ** $p < 0.01$; *** $p < 0.001$; **** $p < 0.0001$).
